# 异基因造血干细胞移植联合CD7 CAR-T细胞疗法治疗T淋巴母细胞性淋巴瘤1例报告及文献复习

**DOI:** 10.3760/cma.j.issn.0253-2727.2023.10.014

**Published:** 2023-10

**Authors:** 祥民 王, 屹 周, 姣丽 张, 泓沅 周, 青 张, 倩 孙, 护君 李, 林艳 徐, 书娜 姚, 志华 姚, 冬梅 闫, 开林 徐, 威 桑

**Affiliations:** 1 徐州医科大学附属医院血液科，徐州医科大学血液病研究所，骨髓干细胞重点实验室，徐州 221002 Department of Hematology, the Affiliated Hospital of Xuzhou Medical University, Blood Diseases Institute, Key Laboratory of Bone Marrow Stem Cell, Xuzhou 221000, China; 2 徐州医科大学附属医院康复科，徐州 221002 Department of Rehabilitation, the Affiliated Hospital of Xuzhou Medical University, Xuzhou, 221000, China; 3 郑州大学附属肿瘤医院淋巴综合内科，郑州 450000 Department of Internal Medicine, Affiliated Cancer Hospital of Zhengzhou University, Henan Cancer Hospital, Zhengzhou 450000, China

T淋巴母细胞性淋巴瘤（T-LBL）是一种高度侵袭性的血液恶性肿瘤，其特征是骨髓和外周血中浸润未成熟的T细胞，常侵犯纵隔，骨髓和淋巴结，预后差[1]。异基因造血干细胞移植（allo-HSCT）可明显改善T-LBL患者的生存[2]，但复发/难治性T-LBL（R/R T-LBL）患者的预后仍然很差[3]。嵌合抗原受体T细胞（CAR-T）疗法作为一种新兴的治疗手段，近年来在复发/难治性血液系统恶性肿瘤患者中取得了令人鼓舞的疗效[4]–[6]。我们采用allo-HSCT联合CD7 CAR-T细胞疗法治疗1例T-LBL获得满意疗效。

## 病例资料

一、一般资料

患者，男，34岁，因“发现双侧肘部、腹股沟处多发肿物1月余”入院。患者2017年11月无意间发现双侧肘部、腹股沟处多发肿物，无疼痛、发热及盗汗，未予特殊重视，1个月后因肿物增大就诊于某肿瘤医院，行左肘部肿物切除术，病理示“T淋巴母细胞性淋巴瘤”，诊断为“T淋巴母细胞淋巴瘤Ⅳa期”。患者在接受6个周期的Hyper-CVAD/MA方案化疗后，获得了完全缓解（CR），继而在2018年5月接受了自体造血干细胞移植。7个月后出现疾病进展（PD），予“培门冬酶+VP”方案化疗达CR后，患者于2019年3月接受了胞姐HLA全相合allo-HSCT。2020年9月再次PD，多线化疗后仍未得到有效控制，为行CAR-T治疗来我院诊治。既往体健，否认慢性病史及手术外伤史。无家族遗传病史及类似病史。免疫组化（左上肢肿物）：EMA（−），CD3（+），CD5（+），CD20（−），CD79a，Pax-5（−），CD43（+），TdT（+），CD99（+），MPO（−），CD10（−），CD30（−），Bc1-2（+），Bc1-6（−），CD7（+），CD56（−），CD4（−），Ki-67（80％+），CD38（+），CD1a（+）。PET-CT：颈部双侧、锁骨上下、纵隔、双侧腋窝、双上臂肌间隙、双肘部皮下、腹膜后、双侧髂外血管走形区及双侧腹股沟多发软组织结节及肿块影，代谢活跃，考虑淋巴瘤浸润，脾大，代谢活跃。骨髓象：淋巴母细胞占10％。

2021年4月，患者在我院接受FC方案（氟达拉滨+环磷酰胺）化疗，随后输注其胞姐来源CD7 CAR-T细胞1×10^6^/kg，CAR-T治疗后发生1级细胞因子释放综合征（CRS），给予支持治疗，未予糖皮质激素和托珠单抗。CAR-T细胞输注后并未观察到临床疗效，PET-CT评估疗效达PD。2021年6月，予改良Bu/Cy方案进行预处理后，行HLA全相合allo-HSCT（供者为胞兄），移植后11 d输注供者来源CD7 CAR-T细胞1×10^6^/kg。移植后16 d粒细胞植入，18 d血小板植入，移植后1个月供者干细胞完全嵌合。CAR-T输注后1个月，PET-CT扫描显示疾病达CR，未发生急性移植物抗宿主病（GVHD）和CRS。患者在此期间出现肺感染，治疗后得到有效控制。半年后，CT扫描发现病情再次PD，再次输注供者来源CD7 CAR- T细胞1×10^6^/kg，治疗后发生1级CRS。2周后CT检查显示纵隔淋巴结明显缩小。2022年7月（allo-HSCT后1年）CT评估病情处于CR状态。

## 讨论

基于CD19的CAR-T细胞治疗在R/R B淋巴细胞白血病患者中达到了70％～90％的CR率[7]–[9]。此外，我们先前的研究表明，抗CD19 CAR-T联合allo-HSCT可使R/R B淋巴细胞白血病患者获得长期存活[10]。CD7是一种跨膜糖蛋白，在90％～96％的正常T细胞中表达[11]，是治疗T细胞淋巴瘤的潜在靶点，靶向CD7的CAR-T细胞被认为在T-ALL中有着较高的CR率[12]–[13]。对于存在骨髓受累的T-LBL，由于正常T细胞和恶性T细胞之间存在共同抗原，目前无法行自体CD7 CAR-T细胞治疗。本例患者单独输注胞姐来源CD7 CAR-T细胞并没有取得令人满意的效果。近年来，CAR-T联合allo-HSCT正逐渐成为一种有效的治疗措施[13]。有研究表明，患者接受CD7 CAR-T后序贯allo-HSCT取得了令人满意的疗效[13]–[14]。然而，预处理和allo-HSCT也可能导致体内CAR-T细胞耗尽，影响CAR-T细胞治疗的长期疗效[14]。如何进一步优化CAR-T联合allo-HSCT策略已成为研究者们需要解决的问题。本例患者allo-HSCT后输注供者来源CD7 CAR-T获得CR疗效，尽管我们在HSCT后使用了GVHD预防性措施。虽然患者在半年后PD，但再次输注供者来源CD7 CAR-T细胞后病情很快得到控制。此外，CD7 CAR-T细胞疗法在本例患者显示出了良好的安全性和耐受性，allo-HSCT后粒细胞和血小板均顺利植入，也未发生急性GVHD。

## References

[1] Jain P, Kantarjian H, Ravandi F (2014). The combination of hyper-CVAD plus nelarabine as frontline therapy in adult T-cell acute lymphoblastic leukemia and T-lymphoblastic lymphoma: MD Anderson Cancer Center experience[J]. Leukemia.

[2] Yasuda S, Najima Y, Konishi T (2021). Outcome of allogeneic hematopoietic stem cell transplantation for T-cell lymphoblastic leukemia/lymphoma: A single-center study[J]. Leuk Res.

[3] Candoni A, Lazzarotto D, Ferrara F (2020). Nelarabine as salvage therapy and bridge to allogeneic stem cell transplant in 118 adult patients with relapsed/refractory T-cell acute lymphoblastic leukemia/lymphoma. A CAMPUS ALL study[J]. Am J Hematol.

[4] Wang Y, Cao J, Gu W (2022). Long-Term Follow-Up of Combination of B-Cell Maturation Antigen and CD19 Chimeric Antigen Receptor T Cells in Multiple Myeloma[J]. J Clin Oncol.

[5] Cao J, Cheng H, Shi M (2019). Humanized CD19-specific chimeric antigen-receptor T-cells in 2 adults with newly diagnosed B-cell acute lymphoblastic leukemia[J]. Leukemia.

[6] Sang W, Shi M, Yang J (2020). Phase II trial of co-administration of CD19- and CD20-targeted chimeric antigen receptor T cells for relapsed and refractory diffuse large B cell lymphoma[J]. Cancer Med.

[7] Park JH, Rivière I, Gonen M (2018). Long-Term Follow-up of CD19 CAR Therapy in Acute Lymphoblastic Leukemia[J]. N Engl J Med.

[8] Shah BD, Bishop MR, Oluwole OO (2021). KTE-X19 anti-CD19 CAR T-cell therapy in adult relapsed/refractory acute lymphoblastic leukemia: ZUMA-3 phase 1 results[J]. Blood.

[9] Qi Y, Zhao M, Hu Y (2022). Efficacy and safety of CD19-specific CAR T cell-based therapy in B-cell acute lymphoblastic leukemia patients with CNSL[J]. Blood.

[10] Chen W, Ma Y, Shen Z (2021). Humanized Anti-CD19 CAR-T Cell Therapy and Sequential Allogeneic Hematopoietic Stem Cell Transplantation Achieved Long-Term Survival in Refractory and Relapsed B Lymphocytic Leukemia: A Retrospective Study of CAR-T Cell Therapy[J]. Front Immunol.

[11] Reinhold U, Abken H, Kukel S (1993). CD7- T cells represent a subset of normal human blood lymphocytes[J]. J Immunol.

[12] Cooper ML, Choi J, Staser K (2018). An “off-the-shelf” fratricide-resistant CAR-T for the treatment of T cell hematologic malignancies[J]. Leukemia.

[13] Pan J, Tan Y, Wang G (2021). Donor-derived CD7 chimeric antigen receptor T cells for T-cell acute lymphoblastic leukemia: first-in-human, phase I trial[J]. J Clin Oncol.

[14] Lu P, Liu Y, Yang J (2022). Naturally selected CD7 CAR-T therapy without genetic manipulations for T-ALL/LBL: first-in-human phase 1 clinical trial[J]. Blood.

